# Nonadherence and Contributing Factors among Ambulatory Patients with Antidiabetic Medications in Adama Referral Hospital

**DOI:** 10.1155/2014/617041

**Published:** 2014-12-03

**Authors:** Belayneh Kefale Gelaw, Abdela Mohammed, Gobezie Temesgen Tegegne, Amsalu Degu Defersha, Muluneh Fromsa, Esayas Tadesse, Thrumurgan Gunasekaran, Mustefa Ahmed

**Affiliations:** Department of Pharmacy, College of Medicine and Health Science, Ambo University, Oromia Region, Ethiopia

## Abstract

The objective of this study was to determine the magnitude of nonadherence and its contributing factors among diabetic patients attending the diabetic clinic in Adama Hospital. *Methods.* This descriptive cross-sectional study was carried out among patients with diabetes mellitus attending the diabetes mellitus clinic of Adama Referral Hospital. Every other patient was selected and data regarding their medication adherence was collected using a structured interview. Data analysis was carried out using SPSS-16. *Result.* The response rate from this study was 98.3%. A total of 270 patients were interviewed; 51.5% were males. A total of 68.1% of the patients included in the study were married. 14% were younger than 40 years, and 50% were between 40 and 60 years. 21.8% of the participants ascribed their nonadherence to forgetting to take their medications. Patients with duration of diabetes ≤5 years (82.07%) were more compliant to their medication than those with >5 years (60.8%), which was found to be statistically significant (*P* = 0.003). Insulin, 47%, and glibenclamide plus metformin, 43.7%, were the most commonly prescribed mono- and combination therapies, respectively. Common comorbid conditions include hypertension, 148 (54.82%), and visual impairment, 89 (32.96%). The proportion of male patients adherent to their antidiabetic medications was found to be lower than 69.78% compared to the female patients (74.81%), but the difference was not statistically significant (*P* > 0.05). *Conclusion.* Most diabetic patients are currently being managed with the most effective available drugs. However the result from this study indicates that the desired blood sugar level could not be controlled and maintained adequately. This was because of poor adherence to the prescribed drug regimen and poor knowledge and practice of successful self-management.

## 1. Introduction

### 1.1. Background

Diabetes mellitus refers to a group of common metabolic disorders that share the phenotype of hyperglycemia. The prevalence of diabetes mellitus is growing rapidly worldwide and is reaching epidemic proportions. It is estimated that there are currently 285 million people with diabetes worldwide and this number is set to increase to 438 million by the year 2030 [1]. Epidemiological data indicate that all nations, rich and poor, are suffering the impact of the diabetes epidemic. The impact is worse in those countries that are socially and economically disadvantaged. In Africans 80% of diabetes patients are undiagnosed. Most of them may be asymptomatic or have mild symptoms which they ignore or attribute to other myths. Some may not present in hospital out of poverty even when symptomatic [2].

Information on chronic complications of diabetes in sub-Saharan Africa is scarce; however, its incidence has gone hand in hand with the growing disease prevalence, demonstrating the importance of assessing complications [3].

Factors contributing to optimum disease management included age, complexity of treatment, duration of disease, and psychosocial issues [4].

Ethiopia is the second most populous country in sub-Saharan Africa where more than 80% of the population lives in the country side. In Ethiopia, national data on prevalence and incidence of diabetes are lacking. However, patient attendance rates and medical admissions in major hospitals are rising. The estimated prevalence of diabetes mellitus (DM) in adult population of Ethiopia is 1.9% [5].

Management of diabetes mellitus involves both pharmacological and nonpharmacological approaches. Nonpharmacological approaches include life style modification, dietary modification, and physical exercise. The pharmacological approach is used when the nonpharmacological approach fails to achieve the desired outcome. Pharmacotherapy for type 2 DM has changed dramatically in the last few years with the addition of several new drug classes and recommendations to achieve more stringent glycemic control. Recently initiation of metformin in all patients with T2D at diagnosis along with appropriate life style modification has been introduced where there is no contraindication. In addition to metformin, OHA, injectable insulin, amylin analogs, and inhaled insulin are other options for treatment of T2D [6]. The choice of therapy for type 1 DM is simple: all patients need insulin. However, how that insulin is delivered to the patient is a matter of considerable practice difference among patients and clinicians [7].

Nonadherence rates are relatively high across disease states, treatment regimens, and age groups. The drop in adherence is noted to be most dramatic after the first six months of therapy among patients with chronic conditions such as diabetes mellitus. A systematic review of studies on adherence to medication among diabetes patients showed that average adherence to oral antidiabetes medications ranges from 36% to 93%, while adherence to other treatment recommendations especially dietary adherence among these patients remains poor. Medication may contribute to nonadherence secondary to its side effects and cost, while poor patient-healthcare provider relationships may also be a major determinant of nonadherence [8].

Poor adherence to medication regimens is common, contributing to substantial worsening of disease, death, and increased healthcare costs. Hence, practitioners should always look for poor adherence and can enhance adherence by emphasizing the value of a patient's regimen, making the regimen simple and customizing the regimen to the patient's lifestyle.

### 1.2. Statement of the Problem

The prevalence of diabetes mellitus is growing rapidly worldwide and is reaching epidemic proportions. Nonadherence, poverty, lack of knowledge, and poor follow-ups are the main factors observed in poor glycemic control. Nonadherence to prescribed medication schedule has been and continues to be a major problem in the world. In chronic disease, it has been described as taking less than 80% of the prescribed treatment. Previous studies have found adherence to diabetes treatment generally to be suboptimal ranging (23%–77%) [9].

In Ethiopia, national data on prevalence and incidence of diabetes are lacking. However, patient attendance rates and medical admissions in major hospitals are rising. The World Health Organization (WHO) estimated the number of diabetic cases in Ethiopia to be 800,000 by the year 2000, and the number is expected to increase to 1.8 million by 2030 [10].

There is a continuing need to routinely assess the likely reasons for nonadherence among patients with diabetes in clinical practice. This is especially important in developing countries such as Ethiopia where economic instability and inadequate access to healthcare facilities might have led to the increased incidence of medication nonadherence. In resource-limited countries like Ethiopia, the preponderance of economic instability, low literacy level, and restricted access to healthcare facilities might have led to the increased incidence of medication nonadherence. To the best of our knowledge, evidence-based research that evaluates medication adherence among patients with diabetes in Ethiopia is scanty.

In addition to this, we have the following.

- Most of the previous studies were done in developed countries, leaving the gaps in knowledge about the prevalence and factors that may be associated with adherence to diabetic patient in Ethiopia.
- Few studies on antidiabetic medication adherence have been reported from Ethiopia.
- The sample size used in some of the studies is very small and the method of selection of participants in some cases has led to highly selective samples that are not representatives of the population from which they are picked.

Therefore the purpose of this study is to fill the gap in knowledge of the adherence and contributing factors and the association between them in diabetic patients in Adama Hospital.

### 1.3. Significance of the Study

Determining the significance of nonadherence and identification of the factors leading to nonadherence to a prescribed treatment through a continued research can assist in planning interventions to overcome the barriers. Hence, this study will be carried out to

- give information on patient nonadherence and related factors that may help in the healthcare system for whom it concerns;
- give information based on the respondent's responses on different aspects of the disease that may help in further study by policy makers and some concerned governmental bodies;
- design an interventional method that can solve problems related to nonadherence;
- give recommendations on how to manage problems associated with nonadherence in diabetic patients;
- help as a baseline for further study on patient's adherence and determine various adherence and nonadherence issues.

### 1.4. Objective

#### 1.4.1. General Objective

The aim of this study was to determine the magnitude of nonadherence and its contributing factors among diabetic patients attending DM clinic in Adama Referral Hospital.

### 1.5. Specific Objectives

**Figure 1:**
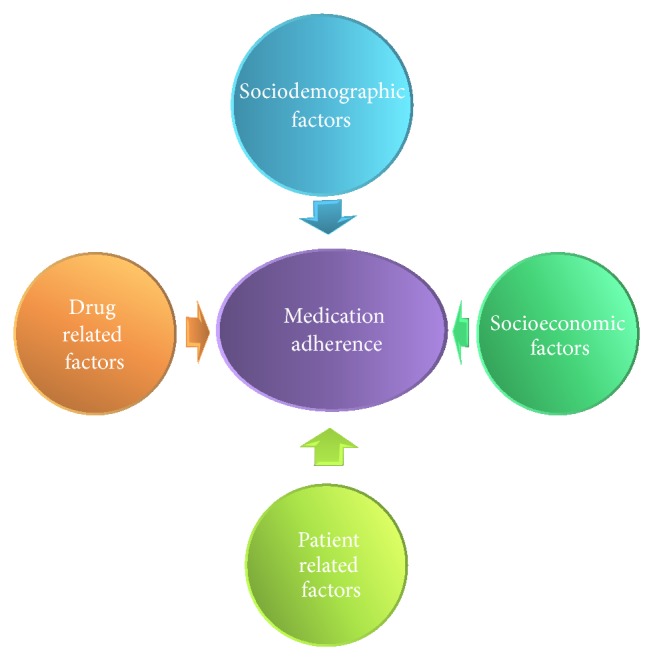
Structural framework.

Specific objectives are as follows:

- to assess adherence to medication among ambulatory patients with diabetes;
- to identify the probable reasons for nonadherence with a view to develop intervention to improve adherence;
- to determine the relationship between nonadherence and various sociodemographic and other drug and patient related factors (Figure 1);
- to describe the prevalence of different perceived problems of respondents with disease or the medication and on the healthcare system;
- to provide the baseline data for future study.

## 2. Study Method

### 2.1. Study Area and Period

The study setting was Adama Referral Hospital, East Showa, Oromia National Regional State, Ethiopia. Adama is located 99 km southeast of Addis Ababa (the capital city of Ethiopia). It was established in 1946 by Italian Missionaries and formerly called “Haile Mariam Mammo Memorial Hospital.” It is a medical college and teaches accelerated medicine, emergency surgery, and anesthesia nurses. The hospital gives services for about 5 million people in east and southern parts of Oromia, Afar, Somali, and Southern Nation, Nationalities, and Peoples' Region (SNNP). Now the hospital has 465 different workers to give different services, of which 194 are administration workers. The other 271 workers are health professionals. There are specialist in different field (23), Practitioners (GP) 36, Nurses (116), Laboratory Workers (20), X-Ray professionals (5), Physiotherapists (2), Sanitarians (2), Biomedical professionals (1), Midwifery (16), Anesthesia nurses (9), Health Officers (9), Psychiatry Nurses (3) And Masters in different fields (14).

The data obtained from the hospital shows that an average of 723 ambulatory diabetes patients attended the clinic for follow-up. There are two formal diabetes clinic days per week “Wednesday and Thursday.” This study was done for a period of one month from 15th of April to 15th of May 2014.

### 2.2. Study Design

A prospective cross-sectional study was conducted at the ambulatory diabetic clinic of Adama Referral Hospital (ARH).

### 2.3. Inclusion Criteria

Inclusion criteria include ambulatory patients who

- are on antidiabetic medications for more than six months;
- consented to participating in the study;
- will attend the diabetic clinic during the study period.

### 2.4. Exclusion Criteria

Exclusion criteria are as follows:

- unconscious patients;
- patient age less than 18 years;
- very ill patients.

### 2.5. Population

#### 2.5.1. Source Population

Source population was diabetic patients being treated at Adama Referral Hospital.

#### 2.5.2. Study Population

Study population included all diabetic patients receiving antidiabetic medication in the ambulatory diabetic clinic during the study period.

### 2.6. Sample Size Determination

The sample size was calculated using single population proportion formula as follows:

(1) $$\begin{array}{c} n=\frac{Z^{2}P\left(1-P\right)}{W^{2}}, \end{array}$$

where *n* = is desired sample size for population >10,000, *Z* = is standard normal duration usually set as 1.96 (which corresponds to 95% confidence level), and *P* = means that we use positive prevalence estimated, to maximize sample size. Negative  prevalence = 1 − 0.5 = 0.5, *W* = degree of accuracy desired (marginal error is 0.05; then the sample size is *n* = (1.96)^2^0.5(1 − 0.5)/(0.05)^2^ = 384.16 = ~384). Since the total population is <10,000, that is, 723, we use the correction formula to determine final sample size:

(2) nf=n1+n/N=3841+384/723=250,

where *N* = final sample size when a population is < 10,000, *n* is initial sample size when the population is >10,000, and *nf* is estimated study population.

Then 10% contingency was added on 250:

(3) $$\begin{array}{c} 250\times 10\%=25\\ nf+\text{contingency}=275. \end{array}$$

### 2.7. Sampling Technique

A systematic random sampling technique was used.

### 2.8. Data Collection Procedure

The study involves cross-sectional interview of consecutive diabetic patients who visit the DM clinic during the study period. The interview was conducted with pretested adherence tool. Patients included in the pretest were subsequently excluded from the study. After the pilot testing, some question-items in the questionnaire were modified and reframed to ensure validity of the instrument (Table 3).

### 2.9. Instruments

The questionnaire, which was the instrument of the study, was pretested on diabetes patients.

This tool consists of information about the sociodemographic characteristics of the respondents (Figure 4), the pattern of drug adherence, and factors contributing to nonadherence. It also consists of information related to drugs prescribed, dose, frequency, and Patients' mean fasting plasma glucose reading at the last clinic visit. Each questionnaire containing 25 questions that took an average of 5 to 10 minutes to fill was used in the interview. It was designed to have two sections; the first section elucidated the sociodemographic characteristics of diabetic patients while the second section contained questions that assess the adherence patterns and the likely reasons for patients' nonadherence to prescribed medications.

### 2.10. Study Variables

#### 2.10.1. Independent Variables

Independent variables are as follows:

- age,
- religion,
- educational level (class year),
- marital status,
- income,
- residence.

#### 2.10.2. Dependent Variables

Dependent variables are as follows:

- knowledge about the medications,
- knowledge about the disease,
- outcomes of treatment with antidiabetic drugs.

### 2.11. Data Analysis

#### 2.11.1. Data Quality Assurance and Interpretation

Data were sorted, coded, and entered into predictive analytics software (PASW) (formerly SPSS) window version 16 for management and analysis. Descriptive statistics including frequency, mean, range, and standard deviation were used to summarize patients' baseline sociodemographic data and evaluate distribution of responses. Correlation and logistic analysis were employed.

## 3. Ethical Considerations

Before data collection to conduct this study ethical approval was obtained from Ambo University College of Medicine and Health Science research team leader and the letter was submitted to Adama Referral Hospital medical director office prior to the beginning of undertaking the study in the area. All the study participants were informed about the purpose of the study; their right to refuse was maintained. Ethical conduct was maintained during data collection and throughout the research process. Verbal consent was obtained from each patient before the interview. Patients were assured of their anonymity. The confidentiality of the data obtained was assured and the name and address of the patient were omitted from the questioner.

## 4. Result

The response rate from this study was 98.3%. A total of 270 patients were interviewed; one hundred thirty-one (48.5%) were females. The mean age for the studied population was 55.11 (SD = 14.24) years (range: 19 to 85 years). The education profile of these patients revealed that 74 (27.4) had no formal or informal education while 99 (36.7%) have secondary or postsecondary education. Sixty-six (24.4%) were retirees from private and public establishments and 33 (12.2%) were government employees. A total of 184 (68.1%) of the patients included in the study were married. Thirty-eight (14%) of the patients were younger than 40 years, one hundred thirty-five (50%) were between 40 and 60 years and 97 (35.9%) were older than 60 years. This and other sociodemographic characteristics are given in Table 1.

**Figure 2:**
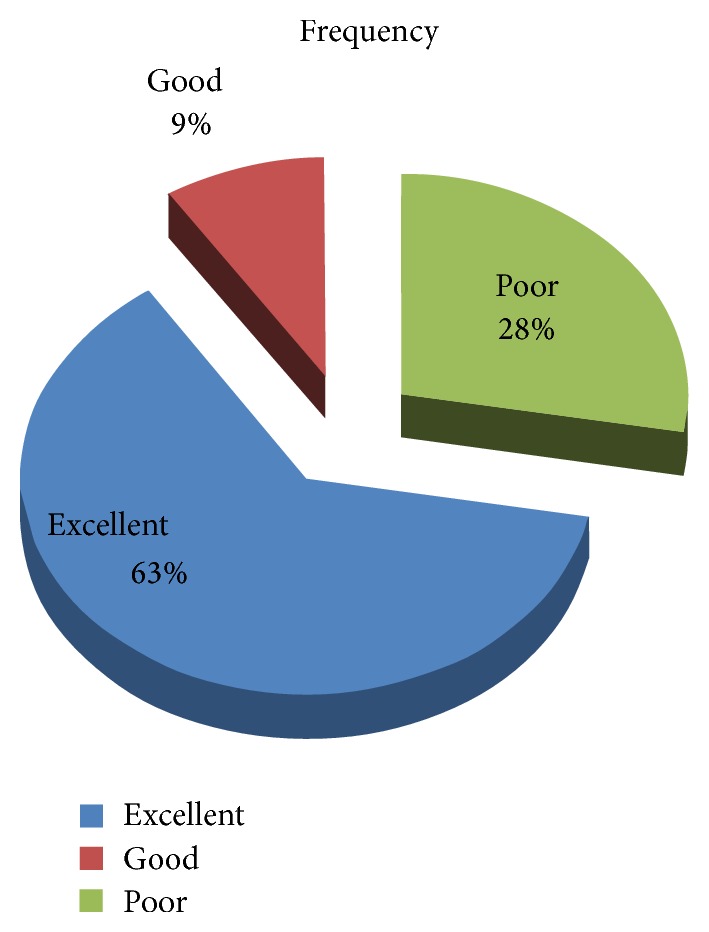
Pattern of adherence, Adama, Ethiopia, 2014.

Approximately 195 (72.2%) of patients self-reported adherence to their antidiabetic drug regimens. In the pattern of drug use, 170 (62.96%) of patients have excellent adherence, 25 (9.26%) have good adherence, and 75 (27.8%) have poor adherence (Table 4 and Figure 2).

**Figure 3:**
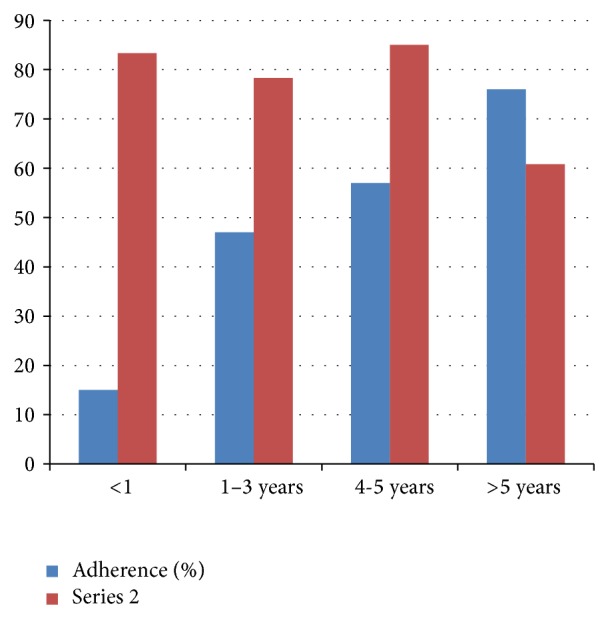
The association between durations of DM adherence, Adama, Ethiopia, 2014 (*P* = 0.003).

**Figure 4:**
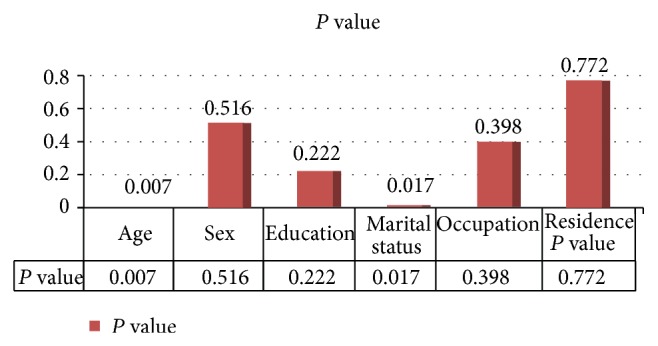
The association between adherence and sociodemographic characteristics, Adama, Ethiopia, 2014.

A total of 59 (21.8%) of the participants ascribed their nonadherence to forgetting to take their medications. Other factors include use of traditional and/or religious medicines, 48 (17.8%), and lack of finances, 39 (14.4%). Of the total population, 248 (91.85%) of the patients reported that they monitored their blood glucose levels monthly at the DM clinic of the hospital on a regular basis. The proportion of male patients adherent to their antidiabetic medications was found to be lower 97 (69.78%) compared to the female patients (74.81%), but the difference was not statistically significant (*P* > 0.05). Adherence to antidiabetic drugs was found to be higher among graduates (postsecondary, e.g., college (80.77%) and university (73.91%)) compared to illiterate and those with up to secondary school education (71.04%), but this finding was not statistically significant (*P* > 0.05). It was also noted that patients with a duration of diabetes ≤5 years (82.07%) were more compliant to their medication than those with diabetes >5 years (60.8%), which was found to be statistically significant (*P* = 0.003) (Table 2 and Figure 3).

Investigation of association between respondents' sociodemographic characteristics and estimates of nonadherence, such as and forgetfulness of medication doses, showed that age and marital status seemed to have statistically significant influence (*P* < 0.05) on respondents' tendencies to have good adherence.

The duration of diabetes from first diagnosis indicates that eighteen (6.7%) had been diagnosed for less than one year, 60 (22.2%) for 1 to 3 years, 67 (22.2%) for 4 to 5 years, and 125 (46.3%) before five years. Comorbid conditions include hypertension, 148 (54.82%), visual impairment, 89 (32.96%), nephropathy, 37 (13.71%), limb paralysis, 30 (11.1%), and no comorbidity, 44 (16.3%). The profile of prescribed anti diabetic medications among the patients indicated that a combination of glibenclamide, and metformine as co-administered products 118 (43.7%) were the most commonly prescribed combination therapy. Insulin alone was used by 127 (47%); glibenclamide alone was used by 19 (7%); and metformin alone was used by 1 (0.4%). Combination of glibenclamide and insulin was used by 4 (1.5%). Only 12 (4.4%) monitor their blood glucose level on regular basis using their glucose measuring device at home. All the respondents, 270 (100%), agree that they needed to continue taking their hypoglycemic medications throughout their lifetime and inappropriate use of medications will lead to development of more problems. Fifty-nine (21.8%) forget to take the prescribed medication(s). Some of the approaches reported to be adopted, once they remembered, included taking the required dose of medication as soon as remembered or skipping it if it is close to the next dose, 66 (24.4%), doubling the next dose to make up for the forgotten dose, 16 (5.9%), and forgetting it completely, 17 (6.3).

## 5. Discussion

The management of diabetes mellitus involves both pharmacologic and nonpharmacologic approaches. For the patient both approaches need a strict compliance to the agreements reached with the physician in order to achieve the desired goals of treatment. Despite this fact most patients were found to be nonadherent to their recommended treatments and this is caused by several factors. As a result assessment of adherence of patients to their respective treatments through continued researches is crucial.

This is a research done on patients with diabetes to evaluate the patients' self-reported adherence to their antidiabetic drug therapy. The prevalence of adherence to antidiabetic medications in this study was 72.2%. In comparison to this finding, two studies conducted in India showed that the patients' self-reported adherence rate to antidiabetic medications was 66.9% and 57.5% [11, 12]. In this regard most patients in the present study are residents in a big city and benefit from the widely disseminated information concerning their disease and directly from their physician.

A systematic review on the compliance to medication among diabetic patients showed that the average compliance to the oral hypoglycemic agents ranged from 36% to 93% [13].

Study from UAE reported a relatively higher overall adherence of 84% [14].

The adherence rates differed across gender and females were more compliant, 74.81%, than males, 69.79%, in the present study. This was in contrast to the result of study from India and UAE [11, 14]. Women spent most of their time at home and they might benefit from this to take their medications as prescribed.

With regard to the educational level, higher adherence rates were noted among graduated patients (diploma), 80.77%, and secondary school patients, 80%, were found to be the most compliant to the prescribed treatment in this study. This was supported by previous researches done in Saudi Arabia and UAE [14, 15]. And it is consistent with the assumption that, as the complexity of the diabetes drug therapy increases, patients are required to understand the prescribed drug therapy to adhere to treatment; hence it would be better understood by those with higher educational profiles. The duration of diabetes plays an important role in management of diabetes. This study showed that most of the patients (53.7%) had a diabetic history of 1–5 years and the longer the duration of diabetes, the lower the rate of adherence (82.07% versus 60.8%) in durations ≤5 years and >6 years, respectively. This finding was consistent with the study from UAE and India indicating a negative relationship between the duration of diabetes and patient adherence to drug therapy [11, 14]. During the early stage of the disease patients tend to be more committed to their disease, but their commitment does not last long since they adapt the burden and deterioration continues.

The most common reasons for nonadherence to medications were modifiable factors that could be overcome by adopting suitable measures. Forgetfulness was the most commonly mentioned reason for noncompliance, similar to the findings of studies from UAE, Nigeria, and India [13, 14, 16]. In contrast, a study from India reported self-decision, 35.08%, as the main causal factor for nonadherence to antidiabetic medications [12]. This barrier can be overcome by assisting patients in organizing their medications with pillboxes and dosing alarms and family members can assist in medication adherence for the elderly and those taking multiple medications.

The high cost of medication is agreed upon by the majority of the patients as the most important reason preventing optimal adherence.

In this study, the main external challenge of adherence is financial problem (61.90%). This is in agreement with study done in Nigeria in which around 66.6%. 37.1% in Ethiopia where the none adherence is due to financial difficulty [8, 17]. Ethiopia is a developing country in which most of the population has a lower income and this is one factor that contributes to the limited health service in general and DM management in particular. The identified causes of nonadherence to taking antidiabetic medications as prescribed were nature of work/busy schedule of work, patient dissatisfaction, cost of drug, and forgetfulness and were found to be 13.85%, 10.77%, 21.54%, and 53.85%, respectively, in this study. Similarly, nonadherence to appointment keeping was caused by forgetfulness, 9.53%, nature of work and busy schedules, 42.86%, travelling away from home, 42.86%, and being intentional, 4.76%. Patients who come from rural areas and those elderly patients who do not have care giver have difficulty of keeping clinic appointments. Similar study identified busy work schedules especially for patients in the working population as one of the reasons why some patients do not take their antidiabetic medications, 16.19% [17].

The majority of the patients were on monotherapy, the same result as a study from Ethiopia [17]. But the monotherapy mostly prescribed in this case was insulin (47%) unlike the above study in which glibenclamide (74.3%) was used. The present study includes both type one and type two diabetes patients and it is not surprising that insulin is used in most patients; that is, it is used in both types I and II (when necessary) and also the prevalence of the types of DM should be considered in these two areas. The most commonly used combination therapy was glibenclamide and metformin (43.7%). This is in agreement with the study in Nigeria that showed the same combination therapy in 36.8% of patients.

The practice of self-monitoring of blood glucose levels by patients is indicative of their commitment to diabetes management. The study showed that 41.1% of the patients had adequate glycemic control and it is consistent with another study which reported adequate glycemic control in 41.8% of type 2 diabetic patients. Although HbA1c is the established gold standard, FPG level is being used to assess and monitor glycemic control in this hospital. The glycosilated hemoglobin (HbA1c) test was not routinely recommended for patients probably on account of the high cost of the test in the hospital or because it may not be part of the established guideline within the hospital.

## 6. Conclusion and Recommendation

### 6.1. Conclusion

This study was able to show the main factors that can undermine the desired outcomes of diabetes pharmacotherapy in diabetic patients by decreasing adherence to their medications. These factors can be patient related such as (forgetfulness, intentional omission of dose) and drug related (cost, side effects, and multiple drug therapy especially in those with comorbidity), all of which are modifiable factors. Most diabetic patients are currently being managed with the most effective available drugs. However as the result from this study indicates the desired blood sugar level could not be controlled and maintained adequately. This was because of poor adherence to the prescribed drug regimen and poor knowledge and practice of successful self-management.

### 6.2. Recommendation

- Adequate, clear, and quality information regarding diabetes and antidiabetic medications should be provided to all diabetic patients in order to make the patient aware of future complications of the disease and the benefits of drug therapy as the factors related to nonadherence in this area are modifiable and associated with low knowledge about the disease and treatment.
- The practice of cost-free medication service to the patients that cannot afford to buy it in this hospital is appreciable as cost of drug is among the factors hindering adherence but the inclusion of other needy patients should be considered since there are still large number of poor patients who are losing hope of their future.
- The role of health professionals at this point should be considerable in providing the most cost-effective, the safest, and the most effective available medication.
- Patients should be encouraged to appropriately use antidiabetic drugs and regular awareness should be created regarding the benefits of using them there by preventing the intentional nonadherence.
- The medication adherence rate in this study was 72.2%. Although the exact estimate of adherence may not be accurately depicted, as this is a small cross-sectional study, future large-scale studies are needed for further understanding of the problem and development of more effective interventions.

## Figures and Tables

**Table 1:** Sociodemographic characteristics of patients, Adama, Ethiopia, 2014.

Variable	Frequency	Percentage	Variable	Frequency	Percentage
Age (years)			Occupation		
18–30	22	8.1	Government		
31–40	16	5.9	Employee	33	12.2
41–50	50	18.5	NGO employee	20	7.4
51–60	85	31.5	Self-employee	75	27.8
>60	97	35.9	Student	54	20
			Housewife	6	2.2
			Retired	66	24.4
			Dependent	16	5.9
Sex			Monthly income		
Female	131	48.5	<500	94	34.8
Male	139	51.5	501–1000	75	27.8
			1001–2000	49	18.1
			>2000	37	13.7
			No income	15	5.6
Marital status			Place of residence		
Single	18	6.7	Rural	51	18.9
Married	184	68.1	Urban	219	81.1
Divorced	33	12.2			
Separated	3	1.1			
Widow/er	32	11.9			
Educational level			Religion		
Never went	74	27.4	Orthodox	149	55.2
Primary school	97	35.9	Muslim	63	23.3
Secondary school	50	18.5	Protestant	43	15.9
Postsecondary	49	18.1	Waqefeta	9	3.3
			Others	6	2.2

**Table 2:** Patients' opinions on factors that prevent optimal medication adherence, Adama, Ethiopia, 2014.

Factors	Frequency	Percentage (%)
Forgetfulness	59	21.8
High cost of the drug	39	14.4
Lack of trust in the efficacy of the drug	9	3.3
Nature or schedule of my work	9	3.3
Traditional and/or religious belief	48	17.8
Side effect of the drug	14	5.2
Feeling better	23	8.5
Feeling worse	9	3.3

**Table 3:** Adherence scores, Adama, Ethiopia, 2014.

Questions	Adherence score (frequency [%])				Mean score
	1	2	3	4	
(1) How often do you forget to take your medicine?	0	6 (2.2%)	53 (19.6%)	211 (78.1%)	3.76
(2) How often do you stop taking your medicine because you feel better?	0	1 (0.4%)	22 (8.1%)	247 (91.5%)	3.91
(3) How often do you stop taking your medicine because you feel worse?	0	0	9 (3.3%)	261 (96.7)	3.97
(4) How often do you stop taking your medicines because you feel they are ineffective?	0	1 (0.4%)	8 (3%)	261 (97.7%)	3.96
(5) How often do you stop taking your medicines because you fear side effects or they have caused side effects?	0	0	14 (5.2%)	256 (94.8%)	3.95
(6) How often do you stop taking your medicine because you are using traditional medicine or religious belief?	0	5 (1.9%)	43 (15.9%)	222 (82.2%)	3.77

Note: adherence scores scales: 4: never; 3: rarely; 2: frequently; 1: daily.

**Table 4:** Frequency distribution of adherers and nonadherers, Adama, Ethiopia, 2014.

Adherence score	Adherence status	Frequency	Percentage (%)
24 (full score)	Adherent	170	62.96
23 (one point missed from question 1)	Adherent	25	9.26
23 (one point missed from another question)	Nonadherent	27	10
20–22	Nonadherent	45	16.67
<20	Nonadherent	3	1.11

Total		270	100

Note: adherers were those who scored a full score of 24 or score of adherence.

## Abbreviations

DM: Diabetes mellitus
OHA: Oral hypoglycemic agents
T2D: Type 2 diabetes
SPSS: Statistical package for social science
CSA: Central Statistics Agency
UAE: United Arab Emirates
ARH: Adama Referral Hospital
FPG: Fasting plasma glucose
HbA1C: Glycated hemoglobin
ADA: American Diabetic Association
SD: Standard deviation
SNNP: Southern Nations, Nationalities, and Peoples’ Region
WHO: World Health Organization
JUSH: Jimma University Specialized Hospital
GP: General practitioner.
